# Current Perspective for Atrial Fibrillation in Patients with Brugada Syndrome: A Comprehensive Review

**DOI:** 10.31083/RCM43820

**Published:** 2025-11-11

**Authors:** Mohammad Iqbal, Rizki Bunawan, Kevin Karim, Giky Karwiky, Chaerul Achmad

**Affiliations:** ^1^Department of Cardiology, Universitas Padjajaran, Dr Hasan Sadikin General Hospital, 40161 Bandung, Indonesia

**Keywords:** Brugada syndrome, atrial fibrillation, *SCN5A*, Implantable Cardioverter-Defibrillator, genetic mutation

## Abstract

Brugada syndrome (BrS) is an inherited cardiac arrhythmia disorder associated with sudden cardiac death (SCD), primarily due to ventricular tachycardia (VT) or ventricular fibrillation (VF). Meanwhile, atrial fibrillation (AF) is becoming increasingly recognized in BrS cases, with a higher prevalence noted among individuals harboring Sodium Voltage-Gated Channel Alpha Subunit 5 (*SCN5A*) variants. However, the prognostic value and management implications of AF in BrS remain unclear. Therefore, this narrative review aims to summarize current evidence on the prevalence, clinical significance, pathophysiological mechanisms, and management of AF in BrS. Relevant studies were identified through systematic searches in the PubMed, EBSCOhost, and Google Scholar databases from inception to July 2025 using Boolean operators with keywords such as “Brugada Syndrome” AND “Atrial Fibrillation”, “Brugada” AND “AF” AND “Management”, and “Brugada” AND “SCN5A” AND “Atrial Arrhythmia”. The bibliographies of the selected articles were further reviewed to identify additional relevant studies. The prevalence of AF among patients with BrS ranged from 6% to 39% across various cohorts. Observational studies demonstrated a higher incidence of *SCN5A*-positive BrS, suggesting that overlapping atrial and ventricular arrhythmogenic substrates exist. Unrecognized BrS in patients presenting with AF may result in inappropriate administration of sodium channel-blocking agents, potentially triggering malignant ventricular arrhythmias. Management strategies include the careful selection of antiarrhythmic drugs, consideration of pulmonary vein isolation (PVI), and implantation of an implantable cardioverter-defibrillator (ICD) device in high-risk cases. Quinidine remains a potential pharmacological option for recurrent ventricular arrhythmias. AF is a relatively common but understudied arrhythmia in BrS. While the direct association of AF with SCD remains uncertain, AF may serve as a marker of a more arrhythmogenic phenotype in BrS. Nonetheless, current guidelines provide limited recommendations for managing AF in this population, underscoring the need for individualized treatment strategies and further research.

## 1. Introduction

Brugada syndrome (BrS) is an inherited arrhythmogenic disorder associated with sudden cardiac death (SCD), most commonly due to ventricular arrhythmias [1]. The predominant arrhythmic events in BrS are ventricular tachycardia (VT) and ventricular fibrillation (VF); however, other rhythm disturbances, including atrial fibrillation (AF), are frequently observed [2]. The presence of AF in BrS has been linked to a more clinical course [1].

AF is frequently reported among individuals carrying *SCN5A* mutations—a gene also implicated in BrS [1, 3]. In patients carrying an *SCN5A* loss-of-function mutation, age-dependent atrial fibrosis and marked conduction slowing—linked to approximately a 50% decrease in atrial Connexin 43 expression—have been reported, indicating a possible common genetic basis for AF and BrS [4].

Multiple studies have identified AF as one of the most common atrial rhythm disturbances in BrS [5]. Reported prevalence of AF among BrS patients in previous investigations spans from 6% to 39% [1]. Although AF is typically attributed to structural heart disease, other etiologic factors should not be overlooked. AF may arise from a combination of inherited and acquired influences affecting autonomic regulation, atrial anatomy, conduction velocity, and possibly other unidentified mechanisms [3].

It has been proposed that disruptions in electrical conduction within the atria and ventricles contribute to disease progression [6]. The risk of SCD increases in patients with recurrent syncope, family history of SCD, autonomic imbalance, atrial remodeling, and conduction delay [6]. However, the prognostic significance of AF in BrS remains uncertain, as most studies have assessed major arrhythmic events (MAEs) rather than direct correlations with SCD [1].

Given the relatively high prevalence of AF in BrS and the unclear mechanisms linking the two, this review aims to provide a comprehensive overview of the epidemiology, clinical significance, pathophysiology, and management of AF in BrS.

## 2. Methods

This narrative review was conducted following a structured search strategy. Literature searches were performed in PubMed, EBSCOhost, and Google Scholar from database inception to July 2025. Boolean operators were used to combine Medical Subject Headings (MeSH) and free-text terms: “Brugada Syndrome” AND “Atrial Fibrillation”, “Brugada” AND “AF” AND “Management”, and “Brugada” AND “*SCN5A*” AND “Atrial Arrhythmia”. No language restrictions were applied. Additional relevant studies were identified by manually reviewing the reference lists of selected articles and recent clinical guidelines. Eligible studies included observational cohorts, clinical trials, case series, and major review articles addressing the epidemiology, pathophysiology, clinical outcomes, or management of AF in BrS. Editorials, correspondence without original data, and studies not addressing AF in BrS were excluded.

## 3. Atrial Fibrillation Coexisting With BrS 

According to a meta-analysis of six studies, AF is correlated with an increased risk in individuals diagnosed with BrS [1]. In a study by Ghaleb *et al*. [7] among 78 AF patients under 45 years of age without prior structural heart disease, 13 (16.7%) exhibited a type 1 Brugada electrocardiogram (ECG) pattern, identified via Holter monitoring or class IA/C antiarrhythmic drugs (IA/C) provocation testing. These patients more frequently reported syncope and a family history of BrS compared with controls [7]. This prevalence is higher than reported in earlier studies, supporting the hypothesis that AF may be linked to latent BrS. In another study of 190 patients with lone AF, 11 demonstrated Brugada ECG patterns following flecainide challenge; none experienced SCD, although three developed VF [8]. In the TETRIS investigation, Conte and colleagues found that of 522 individuals with inherited arrhythmia syndromes (IAS) who also had atrial arrhythmias (AAs), 355 (68%) were identified as having BrS [9]. This substantial proportion underscores the close association between AF and BrS, suggesting that AF is not merely incidental but may serve as a clinical marker of underlying sodium channel dysfunction and atrial conduction abnormalities in BrS patients [10].

The frequent coexistence of AF and BrS suggests a shared arrhythmogenic substrate involving both atrial and ventricular myocardium, potentially increasing arrhythmic risk and influencing clinical management strategies. Up to 30% of BrS patients experience AF without provocation, and its presence is often associated with a less favorable prognosis [11].

## 4. Pathophysiology of Atrial Fibrillation in BrS 

**Fig. 1.**
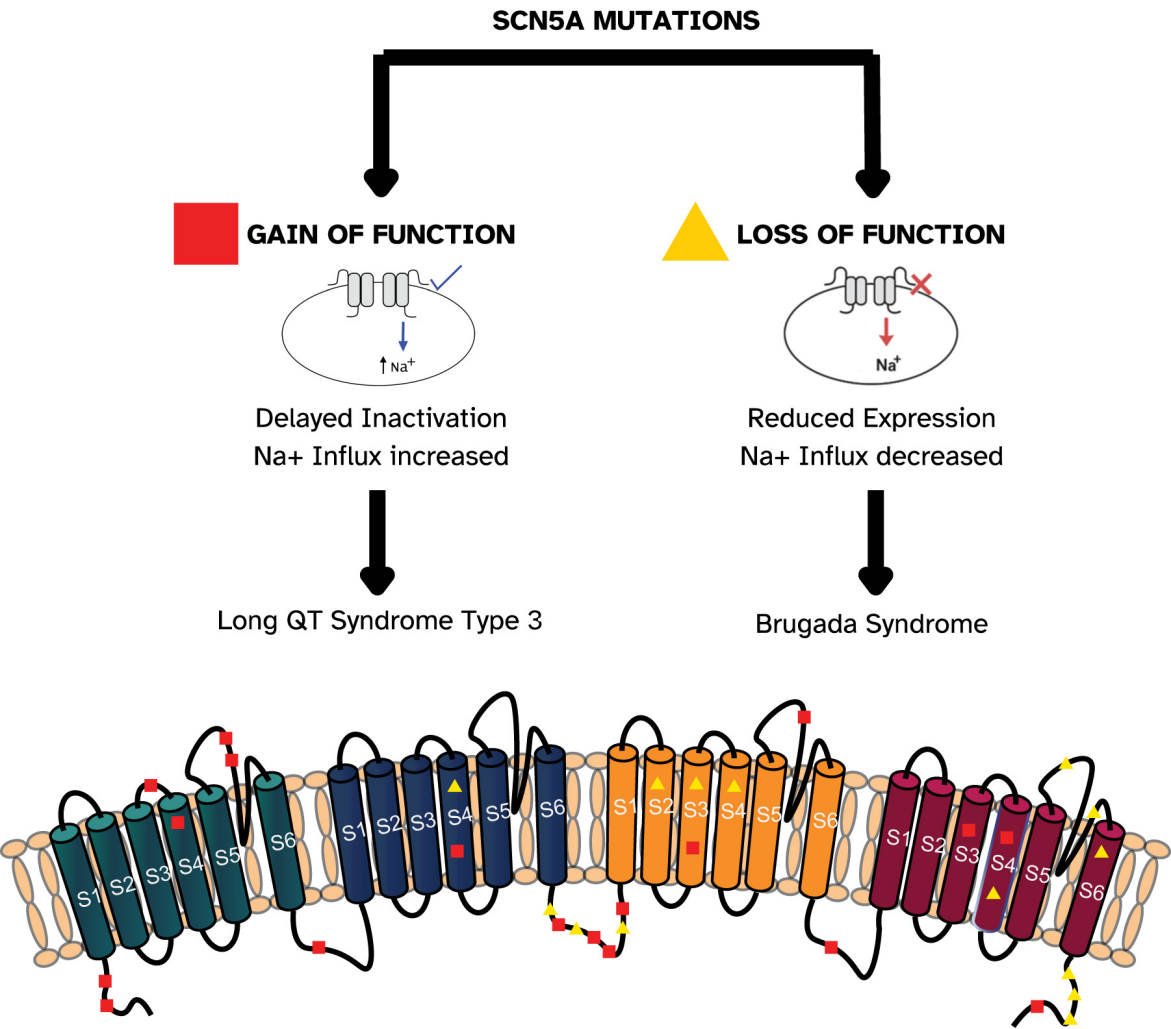
**Schematic representation of Nav1.5 channel domains and examples of *SCN5A* mutations associated with gain- and loss-of-function effects**. The *SCN5A* transcript, consisting of 28 exons, encodes the α-subunit (Nav1.5) of the cardiac sodium channel. Variants in this gene may cause either loss- or gain-of-function by disrupting processes such as transcription, translation, protein folding, trafficking to the membrane, or interactions between Nav1.5 and its regulatory partners. Certain mutations can also alter channel-gating properties [12].

BrS is linked to various genetic abnormalities, most notably in *SCN5A*, which accounts for more than a hundred identified mutations, Fig. 1 (Ref. [12]), present in approximately 20%–30% of patients [13]. Less commonly, variants have been identified in genes affecting sodium currents (Sodium Voltage-Gated Channel Beta Subunit 1 (*SCN1B*), *SCN10A*) and calcium currents (Calcium Voltage-Gated Channel Subunit Alpha1 C (*CACNA1C*), Calcium Voltage-Gated Channel Auxiliary Subunit Alpha 2 delta 1 (*CACNA2D1*), Calcium Voltage-Gated Channel Auxiliary Subunit Beta 2B (*CACNB2B*)) [14]. These mutations typically result in loss-of-function effects, leading to reduced sodium or calcium channel activity and subsequent alterations in cardiac electrophysiology. The functional consequences of these genetic defects have been validated through various experimental approaches. Experimental work on zebrafish also demonstrated that loss-of-function mutations in zebrafish sodium channel orthologs reproduce hallmark BrS features, including slowed atrioventricular conduction, spontaneous arrhythmias, and ST-segment elevation–like ECG changes [15].

The European Society of Cardiology (ESC) guidelines for managing ventricular arrhythmias advise *SCN5A* genetic testing for all individuals with a confirmed diagnosis of Brugada syndrome [14]. In humans, the *SCN5A* gene encodes the α-subunit (Nav1.5) of the cardiac sodium channel, which is essential for depolarization during the action potential [16]. Mutations in *SCN5A* have been implicated in multiple arrhythmic disorders, including long QT syndrome, sinus node dysfunction, cardiac conduction disease, BrS, and AF [17].

Genetic studies have identified 23 genes associated to Brugada syndrome, organized by the ionic currents they regulate: sodium (*INa*) — *SCN5A*, *SCN10A*, Glycerol-3-Phosphate Dehydrogenase 1-Like (*GPD1L*), *SCN1B*, *SCN3B*, RAN Guanine Nucleotide Release Factor (*RANGRF*), *SCN2B*, Plakophilin 2 (*PKP2*), Sarcolemma Associated Protein (*SLMAP*), Fibroblast Growth Factor 12 (*FGF12*); potassium (*IK*) — Potassium Inwardly Rectifying Channel Subfamily J Member 8 (*KCNJ8*), Potassium Voltage-Gated Channel Subfamily H Member 2 (*KCNH2*), Potassium Voltage-Gated Channel Subfamily E Regulatory Subunit 3 (*KCNE3*), Potassium Voltage-Gated Channel Subfamily D Member 3 (*KCND3*), *KCNE5*, *KCND2*, Semaphorin 3A (*SEMA3A*), ATP Binding Cassette Subfamily C Member 9 (*ABCC9*); calcium (*ICa*) — *CACNA1C*, Calcium Voltage-Gated Channel Auxiliary Subunit Beta 2B (*CACNB2B*), *CACNA2D1* [18].

Similarly, AF-related genetic variants include potassium channel genes (*ABCC9*, Hyperpolarization Activated Cyclic Nucleotide Gated Potassium Channel 4 (*HCN4*), Potassium Voltage-Gated Channel Subfamily A Member 5 (*KCNA5*), *KCND3*, *KCNE1*, *KCNE2*, *KCNE3*, *KCNE4*, *KCNE5*, *KCNH2*, Potassium Inwardly Rectifying Channel Subfamily J Member 2 (*KCNJ2*), *KCNJ5*, *KCNJ8*, *Potassium Calcium-Activated Channel Subfamily N Member 3* (*KCNN3*), Potassium Voltage-Gated Channel Subfamily Q Member 1 (*KCNQ1*)) and sodium channel genes (*SCN3B*, *SCN4B*, *SCN5A*, *SCN10A*), and genes involved in gap junction and nuclear pore complex function (Gap Junction Protein Alpha 5 (*GJA5*), *Nucleoporin 155 *(*NUP155*), E169K, Calcium-Sensing Receptor (*CASR*), Paired Like Homeodomain 2 (*PITX2*), Nuclear Receptor Subfamily 4 Group A Member 2 (*NURL1/NR4A2*), Paired Related Homeobox 1 (*PRRX1*), Caveolin 1 (*CAV1*), Cut Like Homeobox 2 (*CUX2*), *Zinc Finger Homeobox 3* (*ZFHX3*)) [19].

Three of the ten sodium channel-related genes (*SCN5A*, *SCN10A*, *SCN3B*) and five of eight potassium channel-related genes (*ABCC9*, *KCNH2*, *KCNE3*, *KCND3*, and *KCNE5*) implicated in BrS are also associated with AF [16]. While no BrS-associated calcium channel genes have been definitively linked to AF, *SCN5A* remains the most extensively studied due to its high prevalence among BrS patients [3, 19].

**Fig. 2.**
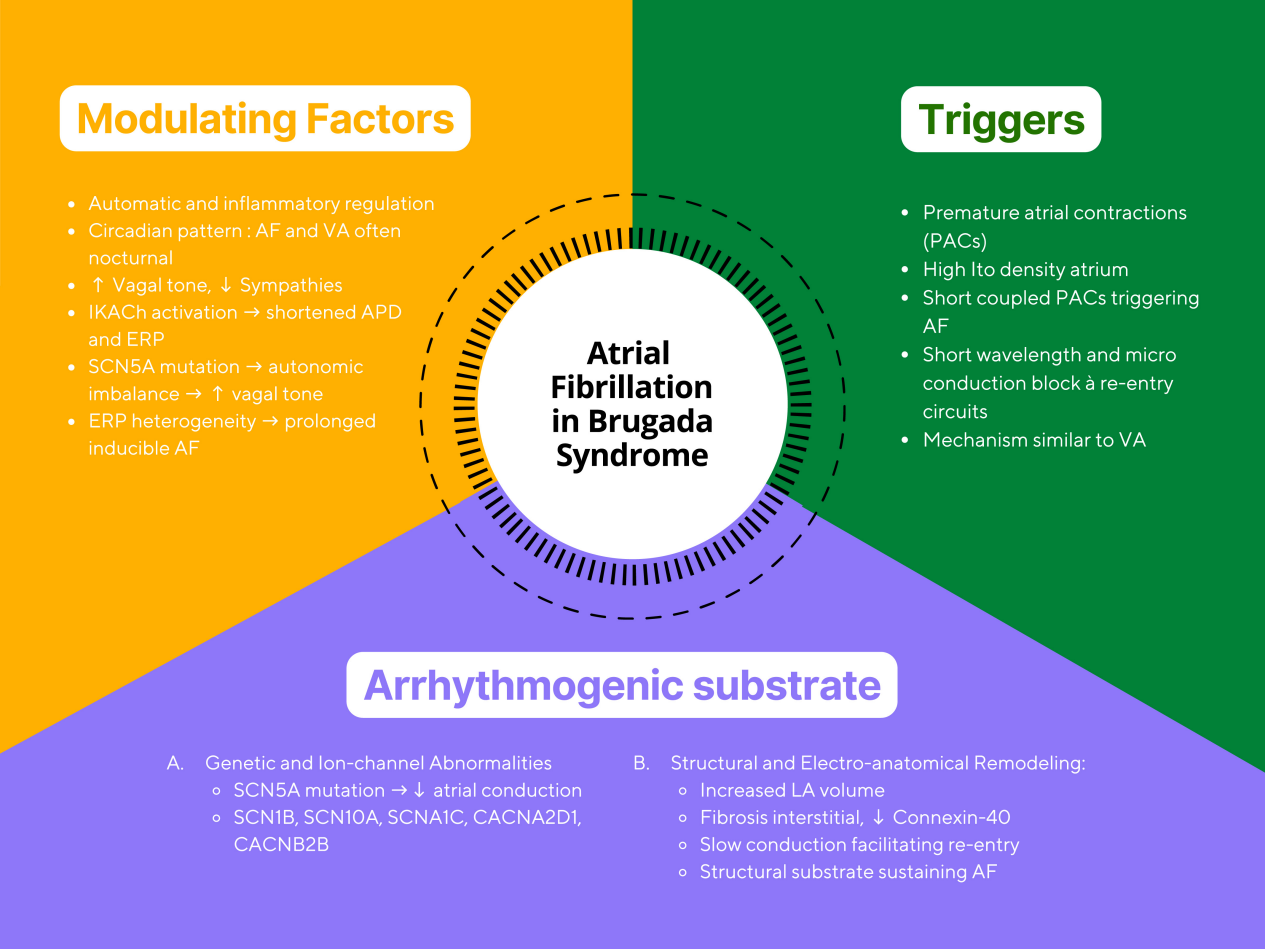
**Conceptual illustration of atrial fibrillation mechanisms in Brugada syndrome**. The figure summarizes the interaction between autonomic triggers, arrhythmogenic substrate, and modulating factors. Abbreviations: AF, atrial fibrillation; BrS, Brugada syndrome; APD, Action Potential Duration; PAC, Premature Atrial Contraction; *Ito*, Transient Outward Potassium Current; SCN5A, Sodium Voltage-Gated Channel Alpha Subunit 5; LA, left atrium; VA, ventricular arrhythmia; IKAch, Acetylcholine-Activated Potassium Current; ERP, Effective Refractory Period.

The pathophysiology of AF in BrS involves an interplay between arrhythmogenic triggers, a vulnerable myocardial substrate, and modulators such as autonomic tone and inflammation (Fig. 2) [4]. AF often follows a circadian rhythm, with most episodes arising at night when vagal influence is greater. Increased vagal stimulation decreases atrial conduction velocity and reduces refractory periods, creating favorable conditions for AF onset [4]. Experimental studies have shown that vagal stimulation can shorten atrial refractory periods and slow conduction, facilitating re-entry. Parasympathetic denervation via ganglionated plexi ablation has been shown to reduce AF by eliminating vagal input, prolonging the effective refractory period (ERP), stabilizing atrial conduction, and suppressing pulmonary vein triggers [20, 21, 22, 23, 24, 25, 26, 27, 28, 29].

Structural atrial abnormalities in BrS and AF patients can delay interatrial conduction, usually serving as a substrate for re-entry [30]. Re-entry requires both an anatomical or functional block and an excitable gap [30]. Slow atrial conduction facilitates re-entry and may explain the higher incidence of tachyarrhythmias in BrS patients [1]. Bradycardia and heightened vagal tone may also reduce calcium influx, contributing to ST-segment elevation and proarrhythmic risk [31].

Abnormalities in cardiac conduction and repolarization are strongly associated with *SCN5A* mutations, which code for the α-subunit (Nav1.5) of the sodium channel [32]. These defects manifest on ECG as right bundle branch block–like patterns accompanied by ST-segment elevation in the right precordial leads [33]. Loss-of-function *SCN5A* mutations impair sodium channel inactivation, disrupt phase 0 depolarization, and alter repolarization. Histopathologic studies have demonstrated subtle myocardial changes in BrS, which may promote slow, progressive remodeling in both the ventricles and atria [33, 34].

Atrial remodeling creates conduction heterogeneity between the atrial myocardium and conduction pathways, acting as both a trigger and perpetuator of AF [35]. One study reported a shortened atrial effective refractory period in the first days of AF, downregulation of L-type calcium channel currents, and upregulation of potassium currents, which shorten the atrial ERP, potentially contributing to the arrhythmogenesis seen in BrS [31].

Autonomic imbalance plays a critical role in AF onset; increased vagal tone slows atrial conduction and shortens refractoriness [36]. Mutations in *SCN5A* may exacerbate intra-atrial conduction delay [32], and patients with both BrS and AF often exhibit marked conduction slowing, suggesting that impaired atrial conduction is a key electrophysiological substrate for AF initiation [30]. Signal-averaged ECG studies have demonstrated prolonged filtered P-wave duration and a higher prevalence of interatrial block in BrS patients with AF, supporting conduction delay as a central mechanism [10].

## 5. Clinical Manifestation of AF in BrS

The clinical presentation of atrial fibrillation of AF ranges widely, from asymptomatic cases to severe outcomes such as cardiogenic shock or stroke [37]. Patients may report mild symptoms, including palpitations, fatigue, reduced exercise tolerance, presyncope, syncope, and dizziness [38].

BrS can be diagnosed in both symptomatic and asymptomatic individuals. Among asymptomatic patients, approximately 63% are diagnosed incidentally. Symptomatic presentations most commonly include syncope, seizures, and VT/VF, which, if sustained, may result in sudden cardiac death [2, 37]. Data from the SABRUS registry indicate that the incidence of syncope and SCD in BrS ranges from 17% to 42%, with SCD most frequently occurring in adult men [37].

Atrial arrhythmias are increasingly recognized in BrS, with prevalence estimates between 6% and 38% [1]. Among these, AF is the most common, affecting approximately 10–20% patients, and is often associated with syncope and an elevated risk of SCD [2]. Genetic analyses have linked AF to *SCN5A* mutations, which are also implicated in BrS, suggesting a possible shared genetic basis. However, this association remains incompletely understood.

## 6. Management of AF in BrS 

Treating AF in patients with BrS poses significant challenges. Due to the pro-arrhythmic potential of sodium channel-blocking antiarrhythmic drugs (AADs), Class IC agents, including flecainide and propafenone, are generally avoided [39]. Furthermore, certain Class III agents, including amiodarone and sotalol, may be hazardous due to their effects on repolarization and potential to induce bradycardia-related arrhythmias [40]. These pharmacological limitations necessitate the investigation of novel therapeutic strategies. In addition to standard treatment strategies, innovative technologies are increasingly shaping AF management. Artificial intelligence (AI) offers significant opportunities across the care spectrum—from early detection and individualized risk assessment to guiding therapeutic choices [41].

### 6.1 Pharmacological Management of AF in BrS

Quinidine, a class IA antiarrhythmic that blocks both *Ito* and *IKr* currents, has demonstrated potential benefits in preventing ventricular arrhythmias and suppressing AF in BrS patients [39, 42, 43]. In a study by Giustetto *et al*. [42], hydroquinidine effectively suppressed AF episodes over 28 months of follow-up in BrS patients. In the cohort studied by Mazzanti *et al*. [44], BrS patients with symptomatic AF treated with quinidine experienced no AF during follow-up, suggesting quinidine may stabilize atrial rhythm while primarily targeting ventricular arrhythmia prevention. Kusano *et al*. [45] reported that two patients with AF and recurrent VF who received quinidine and bepridil experienced no further AF episodes during treatment. Bepridil, a multichannel-blocking AAD, has been shown to reduce both atrial and ventricular arrhythmias in BrS, though its use is limited by risk of QT prolongation and torsades de pointes [40]. Despite limited evidence, bepridil may be considered in highly selected patients under close monitoring.

### 6.2 Role of Catheter Ablation

Pulmonary vein isolation (PVI) has been evaluated as a rhythm control strategy for BrS patients with symptomatic or drug-refractory AF [37]. In one series, freedom from AF after PVI was 76.7%, slightly lower than in the general AF population (80–90%, depending on patient characteristics and ablation techniques) [7]. In BrS, PVI significantly reduces inappropriate implantable cardioverter–defibrillator (ICD) therapies [37]. Similarly, Kitamura *et al*. [46] reported a 92.9% success rate in maintaining sinus rhythm post-PVI and complete elimination of inappropriate ICD therapies after ablation in BrS patients with prior inappropriate shocks. A meta-analysis by Rodríguez-Mañero *et al*. [47] reviewed 49 studies on procedural interventions for AF in BrS, including 49 patients with both BrS and AF, and 39% are still experiencing inappropriate shocks due to AF episodes prior to undergoing PVI [7]. During long-term follow-up after one or more PVI sessions, 91.8% of BrS patients remained free from arrhythmia, and no further inappropriate ICD discharges occurred, supporting catheter ablation as an effective and safe option [7]. Mugnai *et al*. [48] further corroborated these findings, showing a 74% freedom from AF recurrence at three years post-PVI without antiarrhythmic drugs and no major procedural complications. Nonetheless, catheter ablation in BrS requires caution, as underlying structural and electrical atrial abnormalities may contribute to increased post-ablation recurrence risk. Further research is warranted to define optimal ablation strategies and refine patient selection criteria.

### 6.3 ICD in BrS With AF: When is it Relevant?

The role of ICD implantation in BrS patients with concomitant AF remains debated. While ICDs are highly effective in preventing SCD, AF increases the risk of inappropriate shocks due to misclassification of rapid atrial rhythms as ventricular arrhythmias. This may result in patient discomfort, psychological distress, and potential proarrhythmic effects. AF in BrS may also signify more diffuse conduction abnormalities [39, 49]. Inappropriate ICD shocks are frequently triggered by AF. Optimal device programming—such as setting a single, high-rate VF detection zone (≥210–220 bpm) with prolonged detection intervals—can reduce this risk, particularly when monomorphic VT is absent [39]. An atrial lead may be considered in patients experiencing clinically significant bradycardia during beta-blocker therapy [39]. Current guidelines recommend ICD implantation in BrS patients who have survived cardiac arrest or have documented spontaneous sustained VT, regardless of syncope history (Class I) [50]. ICD implantation may also be reasonable in patients with a spontaneous type 1 ECG pattern and syncope suggestive of ventricular arrhythmia (Class IIa), in those with VF inducible by programmed electrical stimulation [50]. The key characteristics and outcomes of the major studies evaluating atrial fibrillation management in BrS are summarized in the Summary Table (Ref. [37, 42, 44, 45, 46, 47, 48]).

**Summary Table.** **Characteristics and outcomes of BrS and AF studies**.

Study	Year	N	BrS + AF	Method	Intervention	Key findings	Outcome
Giustetto *et al*. [42]	2014	560	48 (9%)	Registry-based observational study	Quinidine	Group 1 (AF after BrS diagnosis): younger age, higher spontaneous type 1 ECG, worse prognosis. Group 2 (BrS unmasked by IC drugs): older, better prognosis	AF prevalence is higher than in the general population. HQ is effective and safe for AF prevention
Kusano *et al*. [45]	2008	2	2	Case series	Quinidine 0.3 g oral; Bepridil 100–200 mg/day	No episodes of AF were observed during the therapy	Effective
Mazzanti *et al*. [44]	2019	53	9 (17%)	Prospective Cohort	Quinidine	No recurrent AF episodes	Effective
Bisignani *et al*. [37]	2022	60 (BrS + AF)/60 (control)	60 (50%)	Comparative matched cohort study	PVI	AF freedom rates of 76.7% in BrS vs. 83.3% in control	PVI is less effective in the BrS group
Rodríguez-Mañero *et al*. [47]	2019	49	49 (100%)	Systematic review	PVI	91.8% success rate with PVI; 100% elimination of inappropriate ICD shocks	PVI is highly effective and safe
Kitamura *et al*. [46]	2016	14	14 (100%)	PVI (RF)	PVI (RF)	92.9% had no recurrence of AF	Excellent outcomes with a systematic approach
Mugnai *et al*. [48]	2018	23	13 (56%) underwent PVI	Retrospective observational study	PVI	74% AF-free at 3 years	Good success with both technologies

Abbreviations: AF, atrial fibrillation; BrS, Brugada syndrome; ICD, implantable cardioverter-defibrillator; PVI, pulmonary vein isolation; RF, radiofrequency; VT, ventricular tachycardia; VF, ventricular fibrillation; ECG, electrocardiogram; IC, Ion Channel; HQ, Hydroquinidine.

## 7. Clinical Implications of Unrecognized BrS in AF Patients

One of the most concerning clinical challenges is the unrecognized coexistence of BrS in patients presenting with AF [32]. In such cases, the use of commonly prescribed antiarrhythmic agents for AF management, particularly Class IC drugs such as flecainide or propafenone, may provoke potentially fatal ventricular arrhythmias or sudden cardiac death among individuals with latent Brugada patterns [51]. Class III drugs—such as amiodarone and sotalol—may also exacerbate arrhythmogenic risk through bradycardia-mediated mechanisms or alteration in repolarization [52].

Several case series and registry-based studies have documented instances in which AF was the initial presentation, with BrS remaining undiagnosed until patients experienced ventricular tachyarrhythmias following exposure to sodium channel blockers [7, 35]. Expanding upon the findings of Iqbal *et al*. [53], which indicated a heightened likelihood of sudden cardiac death among individuals with BrS who also exhibited AF, the current investigation examines their clinical profiles, electrophysiological patterns, and potential overlapping mechanisms, aiming to refine both risk assessment and therapeutic approaches. Supporting this concern, observational data from Ghaleb *et al*. [7] demonstrated that among AF patients younger than 45 years without structural heart disease, 16.7% exhibited a type 1 Brugada electrocardiographic pattern, highlighting the underrecognized prevalence of concealed BrS in this population. This risk underscores the importance of identifying Brugada electrocardiogram patterns, whether occurring spontaneously or induced by pharmacological agents, prior to initiating any Class I antiarrhythmic therapy.

Because concealed BrS may remain dormant, particularly in younger patients without structural heart disease, baseline ECG screening should be considered in all new-onset AF cases, especially when AF occurs at a young age or in the presence of a suggestive family history [36]. In selected cases, ajmaline or flecainide challenge testing may be warranted to unmask a Brugada phenotype, provided the procedure is performed in a controlled electrophysiology laboratory setting [54, 55].

To prevent iatrogenic complications, greater clinician awareness is essential, supported by guideline-based precautions before prescribing sodium channel-blocking drugs [14]. Eventually, there is a pressing need to develop standardized screening protocols for BrS in AF patients, particularly in those with early-onset disease or unexplained syncope, and to conduct further research evaluating the cost-effectiveness and clinical outcomes of such screening strategies. Implementation of these measures could substantially reduce the risk of preventable, drug-induced ventricular rhythm disturbances in this vulnerable overlap population.

## 8. Conclusion

A mutation in the *SCN5A* gene alters sodium channel function, resulting in abnormal depolarization and repolarization that can trigger arrhythmias, including AF. Due to the shared genetic basis between AF and BrS through *SCN5A* mutations, the risk of AF in BrS patients is higher than in the general population. AF in the context of BrS may indicate a more severe disease phenotype and may increase the incidence of inappropriate ICD shocks. Both BrS and AF have underlying predispositions involving sodium, potassium, and calcium channels. Several genes associated with BrS have also been implicated in AF, suggesting shared genetic mechanisms. Although BrS is primarily a ventricular arrhythmia, the presence of AF may indicate that the genetic expression predisposing to BrS has already manifested. BrS and AF are thought to arise as phenotypic manifestations of shared genetic mutations, which may explain the genetic link between these two arrhythmic entities. Treatment recommendations for AF in BrS remain limited; however, dual-chamber ICD implantation, quinidine therapy, and PVI have demonstrated some benefit in this patient population.

## References

[1] Kewcharoen J, Rattanawong P, Kanitsoraphan C, Mekritthikrai R, Prasitlumkum N, Putthapiban P (2019). Atrial fibrillation and risk of major arrhythmic events in Brugada syndrome: A meta-analysis. *Annals of Noninvasive Electrocardiology: the Official Journal of the International Society for Holter and Noninvasive Electrocardiology, Inc*.

[2] Brugada J, Campuzano O, Arbelo E, Sarquella-Brugada G, Brugada R (2018). Present Status of Brugada Syndrome: JACC State-of-the-Art Review. *Journal of the American College of Cardiology*.

[3] Sieira J, Brugada P (2017). The definition of the Brugada syndrome. *European Heart Journal*.

[4] Vlachos K, Mascia G, Martin CA, Bazoukis G, Frontera A, Cheniti G (2020). Atrial fibrillation in Brugada syndrome: Current perspectives. *Journal of Cardiovascular Electrophysiology*.

[5] Nagamoto Y, Fujii Y, Morita Y, Ueda Y, Miyake Y, Yamane K (2017). Atrial electrical abnormality in patients with Brugada syndrome assessed by signal-averaged electrocardiography. *Indian Heart Journal*.

[6] Kan KY, Van Wyk A, Paterson T, Ninan N, Lysyganicz P, Tyagi I (2025). Beyond the type 1 pattern: comprehensive risk stratification in Brugada syndrome. *Journal of Interventional Cardiac Electrophysiology: an International Journal of Arrhythmias and Pacing*.

[7] Ghaleb R, Anselmino M, Gaido L, Quaranta S, Giustetto C, Salama MK (2020). Prevalence and Clinical Significance of Latent Brugada Syndrome in Atrial Fibrillation Patients Below 45 Years of Age. *Frontiers in Cardiovascular Medicine*.

[8] Pappone C, Radinovic A, Manguso F, Vicedomini G, Sala S, Sacco FM (2009). New-onset atrial fibrillation as first clinical manifestation of latent Brugada syndrome: prevalence and clinical significance. *European Heart Journal*.

[9] Conte G, Bergonti M, Probst V, Morita H, Tfelt-Hansen J, Behr ER (2024). aTrial arrhythmias in inhEriTed aRrhythmIa Syndromes: results from the TETRIS study. *Europace*.

[10] Bordachar P, Reuter S, Garrigue S, Caï X, Hocini M, Jaïs P (2004). Incidence, clinical implications and prognosis of atrial arrhythmias in Brugada syndrome. *European Heart Journal*.

[11] Morita H, Kusano-Fukushima K, Nagase S, Fujimoto Y, Hisamatsu K, Fujio H (2002). Atrial fibrillation and atrial vulnerability in patients with Brugada syndrome. *Journal of the American College of Cardiology*.

[12] Wilde AAM, Amin AS (2018). Clinical Spectrum of SCN5A Mutations: Long QT Syndrome, Brugada Syndrome, and Cardiomyopathy. *JACC. Clinical Electrophysiology*.

[13] Doundoulakis I, Pannone L, Chiotis S, Della Rocca DG, Sorgente A, Tsioufis P (2024). SCN5A gene variants and arrhythmic risk in Brugada syndrome: An updated systematic review and meta-analysis. *Heart Rhythm*.

[14] Zeppenfeld K, Tfelt-Hansen J, de Riva M, Winkel BG, Behr ER, Blom NA (2022). 2022 ESC Guidelines for the management of patients with ventricular arrhythmias and the prevention of sudden cardiac death: Developed by the task force for the management of patients with ventricular arrhythmias and the prevention of sudden cardiac death of the European Society of Cardiology (ESC) Endorsed by the Association for European Paediatric and Congenital Cardiology (AEPC). *European Heart Journal*.

[15] Verkerk L, Verkerk AO, Wilders R (2024). Zebrafish as a Model System for Brugada Syndrome. *Reviews in Cardiovascular Medicine*.

[16] Veerman CC, Wilde AAM, Lodder EM (2015). The cardiac sodium channel gene SCN5A and its gene product NaV1.5: Role in physiology and pathophysiology. *Gene*.

[17] Deb B, Ganesan P, Feng R, Narayan SM (2021). Identifying Atrial Fibrillation Mechanisms for Personalized Medicine. *Journal of Clinical Medicine*.

[18] Gourraud JB, Barc J, Thollet A, Le Scouarnec S, Le Marec H, Schott JJ (2016). The Brugada Syndrome: A Rare Arrhythmia Disorder with Complex Inheritance. *Frontiers in Cardiovascular Medicine*.

[19] Vutthikraivit W, Rattanawong P, Putthapiban P, Sukhumthammarat W, Vathesatogkit P, Ngarmukos T (2018). Worldwide Prevalence of Brugada Syndrome: A Systematic Review and Meta-Analysis. *Acta Cardiologica Sinica*.

[20] Giannino G, Braia V, Griffith Brookles C, Giacobbe F, D’Ascenzo F, Angelini F (2024). The Intrinsic Cardiac Nervous System: From Pathophysiology to Therapeutic Implications. *Biology*.

[21] Kim MY, Coyle C, Tomlinson DR, Sikkel MB, Sohaib A, Luther V (2022). Ectopy-triggering ganglionated plexuses ablation to prevent atrial fibrillation: GANGLIA-AF study. *Heart Rhythm*.

[22] Stavrakis S, Po S (2017). Ganglionated Plexi Ablation: Physiology and Clinical Applications. *Arrhythmia & Electrophysiology Review*.

[23] Pokushalov E, Romanov A, Artyomenko S, Turov A, Shugayev P, Shirokova N (2010). Ganglionated plexi ablation for longstanding persistent atrial fibrillation. *Europace*.

[24] Kumar A, Shariff M, Pachon JC, Acosta JCZ, DeSimone CV, Stulak J (2025). A comparative meta-analysis of addition of ganglionic plexus ablation versus no ganglionic plexus ablation to pulmonary vein isolation for atrial fibrillation. *Journal of Interventional Cardiac Electrophysiology: an International Journal of Arrhythmias and Pacing*.

[25] Younes H, Ademi B, Tsakiris E, Feng H, Pandey AC, Mekhael M (2025). Direct-to-catheter ablation versus second line catheter ablation for persistent atrial fibrillation: Effect on arrhythmia recurrence, AF burden, early left atrium remodeling and quality of life. *Journal of Interventional Cardiac Electrophysiology: an International Journal of Arrhythmias and Pacing*.

[26] Packer DL, Mark DB, Robb RA, Monahan KH, Bahnson TD, Poole JE (2019). Effect of Catheter Ablation vs Antiarrhythmic Drug Therapy on Mortality, Stroke, Bleeding, and Cardiac Arrest Among Patients With Atrial Fibrillation: The CABANA Randomized Clinical Trial. *JAMA*.

[27] Miyamoto K, Kanaoka K, Yodogawa K, Fujimoto Y, Fukunaga H, Asano S (2025). Cryoballoon vs radiofrequency ablation in persistent atrial fibrillation: the CRRF-PeAF trial. *European Heart Journal*.

[28] Maurhofer J, Kueffer T, Madaffari A, Stettler R, Stefanova A, Seiler J (2024). Pulsed-field vs. cryoballoon vs. radiofrequency ablation: a propensity score matched comparison of one-year outcomes after pulmonary vein isolation in patients with paroxysmal atrial fibrillation. *Journal of Interventional Cardiac Electrophysiology: an International Journal of Arrhythmias and Pacing*.

[29] Reddy VY, Gerstenfeld EP, Natale A, Whang W, Cuoco FA, Patel C (2023). Pulsed Field or Conventional Thermal Ablation for Paroxysmal Atrial Fibrillation. *The New England Journal of Medicine*.

[30] Andreasen L, Nielsen JB, Darkner S, Christophersen IE, Jabbari J, Refsgaard L (2014). Brugada syndrome risk loci seem protective against atrial fibrillation. *European Journal of Human Genetics: EJHG*.

[31] Blok M, Boukens BJ (2020). Mechanisms of Arrhythmias in the Brugada Syndrome. *International Journal of Molecular Sciences*.

[32] Radford D, Chou OH, Bazoukis G, Letsas K, Liu T, Tse G (2022). Electrocardiographic features in SCN5A mutation-positive patients with Brugada and early repolarization syndromes: a systematic review and meta-analysis. *International Journal of Arrhythmia*.

[33] Li KHC, Lee S, Yin C, Liu T, Ngarmukos T, Conte G (2020). Brugada syndrome: A comprehensive review of pathophysiological mechanisms and risk stratification strategies. *International Journal of Cardiology. Heart & Vasculature*.

[34] Aziz HM, Zarzecki MP, Garcia-Zamora S, Kim MS, Bijak P, Tse G (2022). Pathogenesis and Management of Brugada Syndrome: Recent Advances and Protocol for Umbrella Reviews of Meta-Analyses in Major Arrhythmic Events Risk Stratification. *Journal of Clinical Medicine*.

[35] Sheikh AS, Ranjan K (2014). Brugada syndrome: a review of the literature. *Clinical Medicine (London, England)*.

[36] Pappone C, Santinelli V (2019). Brugada Syndrome: Progress in Diagnosis and Management. *Arrhythmia & Electrophysiology Review*.

[37] Bisignani A, Conte G, Pannone L, Sieira J, Del Monte A, Lipartiti F (2022). Long-Term Outcomes of Pulmonary Vein Isolation in Patients With Brugada Syndrome and Paroxysmal Atrial Fibrillation. *Journal of the American Heart Association*.

[38] Tzeis S, Gerstenfeld EP, Kalman J, Saad E, Shamloo AS, Andrade JG (2024). 2024 European Heart Rhythm Association/Heart Rhythm Society/Asia Pacific Heart Rhythm Society/Latin American Heart Rhythm Society expert consensus statement on catheter and surgical ablation of atrial fibrillation. *Journal of Interventional Cardiac Electrophysiology: an International Journal of Arrhythmias and Pacing*.

[39] Hindricks G, Potpara T, Dagres N, Arbelo E, Bax JJ, Blomström-Lundqvist C (2021). 2020 ESC Guidelines for the diagnosis and management of atrial fibrillation developed in collaboration with the European Association for Cardio-Thoracic Surgery (EACTS): The Task Force for the diagnosis and management of atrial fibrillation of the European Society of Cardiology (ESC) Developed with the special contribution of the European Heart Rhythm Association (EHRA) of the ESC. *European Heart Journal*.

[40] Tisdale JE, Chung MK, Campbell KB, Hammadah M, Joglar JA, Leclerc J (2020). Drug-Induced Arrhythmias: A Scientific Statement From the American Heart Association. *Circulation*.

[41] Liang H, Zhang H, Wang J, Shao X, Wu S, Lyu S (2024). The Application of Artificial Intelligence in Atrial Fibrillation Patients: From Detection to Treatment. *Reviews in Cardiovascular Medicine*.

[42] Giustetto C, Cerrato N, Gribaudo E, Scrocco C, Castagno D, Richiardi E (2014). Atrial fibrillation in a large population with Brugada electrocardiographic pattern: prevalence, management, and correlation with prognosis. *Heart Rhythm*.

[43] Al-Khatib SM, Stevenson WG, Ackerman MJ, Bryant WJ, Callans DJ, Curtis AB (2018). 2017 AHA/ACC/HRS Guideline for Management of Patients With Ventricular Arrhythmias and the Prevention of Sudden Cardiac Death: A Report of the American College of Cardiology/American Heart Association Task Force on Clinical Practice Guidelines and the Heart Rhythm Society. *Circulation*.

[44] Mazzanti A, Tenuta E, Marino M, Pagan E, Morini M, Memmi M (2019). Efficacy and limitations of quinidine in patients with Brugada syndrome. *Circulation: Arrhythmia and Electrophysiology*.

[45] Kusano KF, Taniyama M, Nakamura K, Miura D, Banba K, Nagase S (2008). Atrial fibrillation in patients with Brugada syndrome relationships of gene mutation, electrophysiology, and clinical backgrounds. *Journal of the American College of Cardiology*.

[46] Kitamura T, Fukamizu S, Kawamura I, Hojo R, Aoyama Y, Komiyama K (2016). Long-term efficacy of catheter ablation for paroxysmal atrial fibrillation in patients with Brugada syndrome and an implantable cardioverter-defibrillator to prevent inappropriate shock therapy. *Heart Rhythm*.

[47] Rodríguez-Mañero M, Kreidieh B, Valderrábano M, Baluja A, Martínez-Sande JL, García-Seara J (2019). Ablation of atrial fibrillation in patients with Brugada syndrome: A systematic review of the literature. *Journal of Arrhythmia*.

[48] Mugnai G, Hünük B, Ströker E, Ruggiero D, Coutino-Moreno HE, Takarada K (2018). Long-term outcome of pulmonary vein isolation in patients with paroxysmal atrial fibrillation and Brugada syndrome. *Europace*.

[49] Watanabe E, Okajima K, Shimane A, Ozawa T, Manaka T, Morishima I (2017). Inappropriate implantable cardioverter defibrillator shocks-incidence, effect, and implications for driver licensing. *Journal of Interventional Cardiac Electrophysiology: an International Journal of Arrhythmias and Pacing*.

[50] Priori SG, Wilde AA, Horie M, Cho Y, Behr ER, Berul C (2013). Executive summary: HRS/EHRA/APHRS expert consensus statement on the diagnosis and management of patients with inherited primary arrhythmia syndromes. *Europace*.

[51] Brodie OT, Michowitz Y, Belhassen B (2018). Pharmacological Therapy in Brugada Syndrome. *Arrhythmia & Electrophysiology Review*.

[52] Conte G, Sieira J, Sarkozy A, de Asmundis C, Di Giovanni G, Chierchia GB (2013). Life-threatening ventricular arrhythmias during ajmaline challenge in patients with Brugada syndrome: incidence, clinical features, and prognosis. *Heart Rhythm*.

[53] Iqbal M, Lesmana MA, Putra IC, Karwiky G, Achmad C, Goenawan H (2024). Implications of Associated Atrial Fibrillation in Brugada Syndrome for Sudden Cardiac Death–A Case Series Analysis. *The American Journal of Case Reports*.

[54] Wilde AAM, Amin AS, Morita H, Tadros R (2023). Use, misuse, and pitfalls of the drug challenge test in the diagnosis of the Brugada syndrome. *European Heart Journal*.

[55] Behr ER, Winkel BG, Ensam B, Alfie A, Arbelo E, Berry C (2025). The diagnostic role of pharmacological provocation testing in cardiac electrophysiology: a clinical consensus statement of the European Heart Rhythm Association and the European Association of Percutaneous Cardiovascular Interventions (EAPCI) of the ESC, the ESC Working Group on Cardiovascular Pharmacotherapy, the Association of European Paediatric and Congenital Cardiology (AEPC), the Paediatric & Congenital Electrophysiology Society (PACES), the Heart Rhythm Society (HRS), the Asia Pacific Heart Rhythm Society (APHRS), and the Latin American Heart Rhythm Society (LAHRS). *Europace*.

